# Anatomical labeling of intracranial arteries with deep learning in patients with cerebrovascular disease

**DOI:** 10.3389/fneur.2022.1000914

**Published:** 2022-10-17

**Authors:** Adam Hilbert, Jana Rieger, Vince I. Madai, Ela M. Akay, Orhun U. Aydin, Jonas Behland, Ahmed A. Khalil, Ivana Galinovic, Jan Sobesky, Jochen Fiebach, Michelle Livne, Dietmar Frey

**Affiliations:** ^1^Charité Lab for Artificial Intelligence in Medicine, Charité Universitätsmedizin Berlin, Berlin, Germany; ^2^Quality | Ethics | Open Science | Translation Center for Transforming Biomedical Research, Berlin Institute of Health (BIH), Charité Universitätsmedizin Berlin, Berlin, Germany; ^3^Faculty of Computing, Engineering and the Built Environment, School of Computing and Digital Technology, Birmingham City University, Birmingham, United Kingdom; ^4^Centre for Stroke Research Berlin, Charité Universitätsmedizin Berlin, Berlin, Germany; ^5^Department of Neurology, Max Planck Institute for Human Cognitive and Brain Sciences, Leipzig, Germany; ^6^Mind, Brain, Body Institute, Berlin School of Mind and Brain, Humboldt-Universität Berlin, Berlin, Germany; ^7^Biomedical Innovation Academy, Berlin Institute of Health, Berlin, Germany; ^8^Department of Neurology, Johanna-Etienne-Hospital, Neuss, Germany

**Keywords:** anatomical labeling, intracranial arteries, cerebrovascular, stroke, deep learning, UNET

## Abstract

Brain arteries are routinely imaged in the clinical setting by various modalities, e.g., time-of-flight magnetic resonance angiography (TOF-MRA). These imaging techniques have great potential for the diagnosis of cerebrovascular disease, disease progression, and response to treatment. Currently, however, only qualitative assessment is implemented in clinical applications, relying on visual inspection. While manual or semi-automated approaches for quantification exist, such solutions are impractical in the clinical setting as they are time-consuming, involve too many processing steps, and/or neglect image intensity information. In this study, we present a deep learning-based solution for the anatomical labeling of intracranial arteries that utilizes complete information from 3D TOF-MRA images. We adapted and trained a state-of-the-art multi-scale Unet architecture using imaging data of 242 patients with cerebrovascular disease to distinguish 24 arterial segments. The proposed model utilizes vessel-specific information as well as raw image intensity information, and can thus take tissue characteristics into account. Our method yielded a performance of 0.89 macro F1 and 0.90 balanced class accuracy (bAcc) in labeling aggregated segments and 0.80 macro F1 and 0.83 bAcc in labeling detailed arterial segments on average. In particular, a higher F1 score than 0.75 for most arteries of clinical interest for cerebrovascular disease was achieved, with higher than 0.90 F1 scores in the larger, main arteries. Due to minimal pre-processing, simple usability, and fast predictions, our method could be highly applicable in the clinical setting.

## Introduction

Intracranial arteries are complex vascular structures. The structural diversity of arteries is not only dependent on individual anatomy but is changing over time owing to physiological and pathophysiological processes (1). One of the most common reasons is cerebrovascular disease, which is characterized by changes in the vasculature presenting as occlusion or stenosis of blood vessels (2). One such cerebrovascular disease is stroke, a widespread and devastating disease. Arterial brain vessels are routinely depicted in the clinical setting by various modalities, e.g., time-of-flight magnetic resonance angiography (TOF-MRA), contrast-enhanced magnetic resonance angiography (CE-MRA), or computed tomography angiography (CTA). These imaging techniques have great potential to be utilized for the diagnosis, prognostication of disease, and monitoring of disease progression (3). Moreover, from a clinical perspective, they play a crucial role in the assessment of eligibility and response to treatment or successful invasive interventions, such as thrombectomy or coiling. For this purpose, however, the information within the images needs to be extracted and quantified. Currently, only qualitative assessment is implemented in clinical applications, relying on visual inspection, which is heavily affected by reader experience and gives rise to limitations, such as interrater variability or differentiation between natural variation vs. pathology. While manual or semi-automated approaches for quantification exist, such solutions are impractical in the clinical setting as they are time-consuming and/or involve too many processing steps. Thus, there is a strong need for the automation of vessel quantification in the clinical setting.

In the context of arterial brain vessels, automation is based on two main steps. First, brain vessels have to be extracted in the 3D space, i.e., segmentation (4). Second, standard anatomical labels need to be assigned to segments of the segmented vessel tree, e.g., a certain segment is identified as belonging to the internal carotid artery (ICA). In the context of cerebrovascular disease, this allows for the exact localization of stenosis, vessel impediments or occlusions, facilitating treatment decisions, and assisting intervention.

Importantly, automated tools need to be computationally light and easily deployed on clinical workstations. The ease of deployment in the software environments of workstations depends on the number of software components (e.g., for pre-processing steps) and how many of them are proprietary. In addition, outputs have to be accurate, robust to pathological deformations or artifacts, and easy to read by clinicians. In the context of segmentation, such models already exist, e.g., Hilbert et al.(5). However, we are not aware of any automated tool for anatomical labeling tailored to the clinical setting with the above specifications.

While anatomical labeling of arteries has been studied in the research setting, most works have not emphasized the aspect of usability in the clinical setting. Existing solutions can be categorized into two main groups, such as location-based approaches and graph-based approaches.

Location-based methods mainly promote classic medical image processing techniques, such as co-registered segment atlases, maps, or reference points to determine the labels of the overlaid arteries (6–8). Consequently, their application and accuracy highly depend on (1) atlas creation or reference point delineation, which demands domain expertise and extensive data, as well as (2) co-registration; a potentially unstable mechanism in case of pathologies and image artifacts.

In case of graph-based methods, the vasculature is represented as a continuous relational graph of nodes and edges, nodes representing all unique points in arterial centerlines and edges connecting them (9–13). Segments of the intracranial arteries are defined between bifurcations, i.e., between nodes that are connected to more than two nodes. Frequently, nodes within a segment—with two connected edges—are neglected to reduce the computational burden due to the size of the graph (11). This means, however, that (1) for labeling of an arterial segment, only bifurcation and ending nodes are taken into account, limiting the method's ability to identify segment-specific abnormalities and (2) methods are bound to *a priori* extraction of bifurcations. Published works differ in the methodology of assigning labels to the nodes, commonly realized by probabilistic modeling. The main disadvantage of graph-based approaches is the need for a connected vascular tree. In the case of intracranial arteries, this can be a major issue. Besides natural variations, e.g., the Circle of Willis, most cerebrovascular disorders cause structural abnormalities of the vasculature due to severe stenosis or occlusion (14). This can lead to missing arteries in imaging—i.e., disconnected vessel tree—and translate to lower performance in patients where the potential clinical impact would be highest.

Moreover, both directions exclusively focus on features crafted from vessels or arterial centerlines, such as coordinates, distances, direction, average radius, or length of segments. Given that vessels comprise only about 1% of the brain volume (15), 99% of the information in a 3D scan, e.g., tissue characteristics around vessels, is not utilized. Such information can play an important role in the localization and facilitate better recognition of pathological variations due to their reflective effect on surrounding tissue.

As an alternative, we present a novel deep learning (DL)-based artificial intelligence (AI) solution for anatomical labeling of intracranial arteries that utilizes holistic information from 3D TOF-MRA images. We adapted a multi-scale Unet-based architecture, called BRAVE-NET, which has demonstrated state-of-the-art performance in brain vessel segmentation. Our model was trained on 242 patients with cerebrovascular disease derived from two datasets. Model input consisted of raw image intensities as image input and artery centerlines with radius as vessel-specific input. Our proposed pipeline relies on minimal pre-processing compared to pipelines in the literature and could be deployed on any clinical workstation or scanner. We analyzed a total number of 24 arterial segments and achieved excellent performance on intracranial arteries of major clinical interest in cerebrovascular disease and promisingly high performance on smaller artery branches.

Our contribution can be summarized as:

- Investigate the performance of image intensity and vessel-specific information for anatomical labeling of intracranial arteries.
- Applying deep learning to label a large number of arterial segments of intracranial arteries.
- Reducing necessary pre-processing steps to the minimum.
- Demonstrating utilization of bifurcation information for image intensity-based labeling of intracranial arteries through segment washing.
- Showcasing adaptability and versatility of the BRAVE-NET vessel segmentation architecture.

## Materials and methods

### Data

#### Patients

Retrospective data from the PEGASUS (16) and 1000Plus (17) studies were analyzed in this study. The PEGASUS study enrolled patients with steno-occlusive cerebrovascular disease with stenosis and/or occlusion of the middle cerebral artery or internal carotid artery in at least 70% of the cases. Of the total of 82 patients, four patients did not have TOF-MRA imaging and six patients were excluded due to low-quality imaging due to patient motion, resulting in 72 patients included in our analysis. The 1000Plus study enrolled patients with acute ischemic stroke within 24 h after symptom onset. In an ongoing process, raw imaging data are processed *via* semi-manual segmentation and anatomical labeling ground-truth creation. This is done according to the study-ID of the patients without any bias toward patient criteria. At the time of this study, 170 patients were fully processed and ready for analysis. Thus, in total, we included 242 patients in our analysis.

Both studies were carried out in accordance with the recommendations of the authorized institutional ethical review board of Charité Universitätsmedizin Berlin. All patients gave written informed consent in accordance with the Declaration of Helsinki.

#### Accessibility

Due to data privacy laws, the imaging data used in this study cannot be currently published. Implementation of the proposed network, as well as the training, prediction, and evaluation frameworks can be found on GitHub at https://github.com/prediction2020/vessel_annotation.

#### Image sequence specifications

Time-of-flight (TOF) magnetic resonance angiography (MRA) images from two datasets were used to train models. TOF-MRA is one of the most important methods for non-contrast neurovascular and peripheral MRA and is thus frequently used in cerebrovascular patients. In both PEGASUS and 1000Plus studies, TOF imaging was acquired with a Magnetom Trio 3T whole-body system (Siemens Healthcare, Erlangen, Germany) using a 12-channel receive radiofrequency (RF) coil (Siemens Healthcare) tailored for head imaging. Voxel sizes were 0.52 × 0.52 × 0.65 mm, matrix sizes 312 × 384 × 127 voxels, TR/TE = 22/3.86 ms, respectively, time of acquisition 3:50 (min:s) and flip angles = 18 degrees.

#### Data preparation

**Figure 1:**
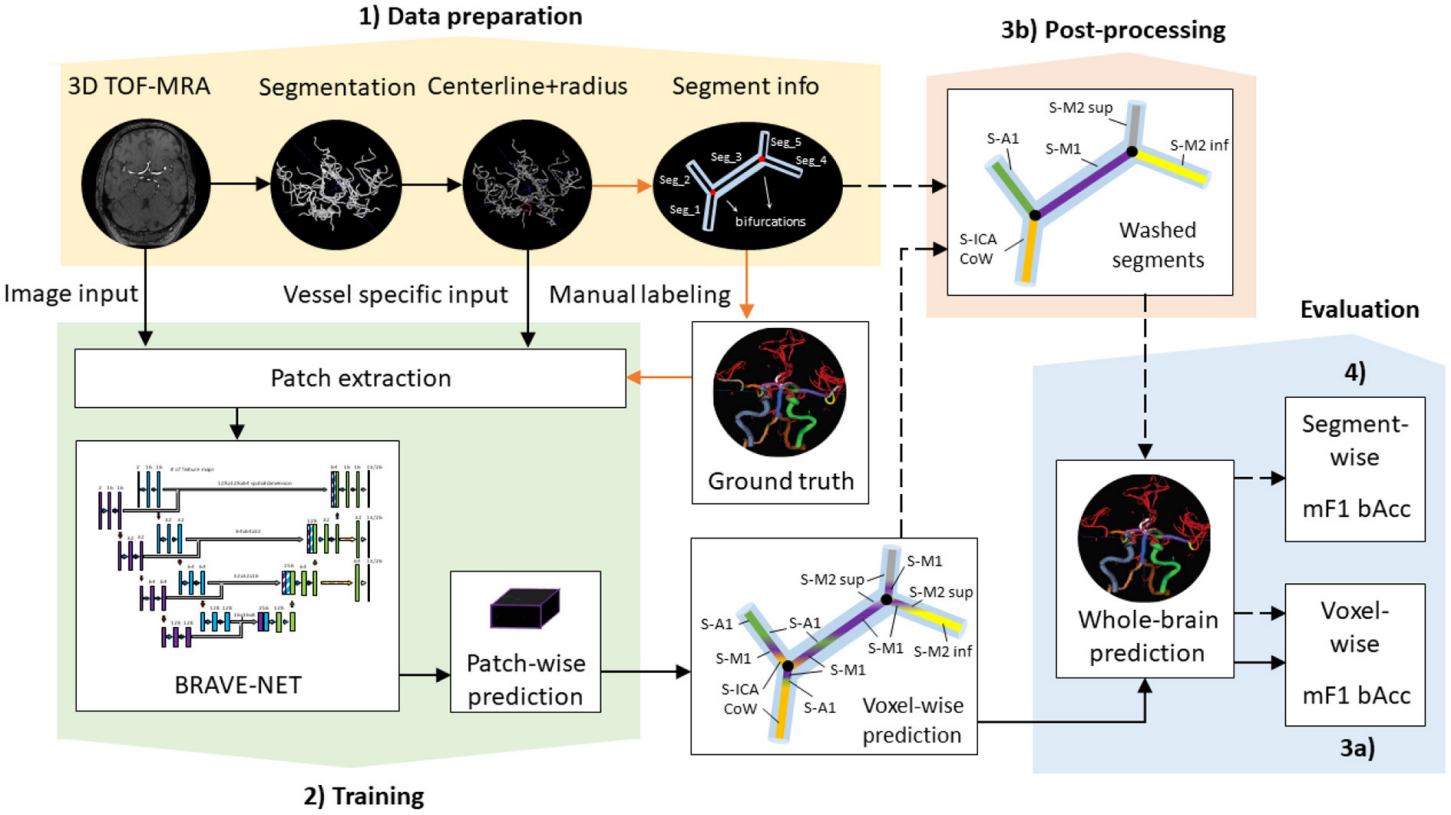
Proposed data processing pipeline. Black arrows show steps necessary for retrieving (filled) and optionally post-processing (dashed) predictions, while orange arrows highlight steps only required for ground-truth creation—i.e., not for making predictions. (1) Data preparation includes processing steps for the extraction of model input such as segmentation and centerline extraction, as well as the extraction of segment information necessary for ground-truth creation. (2) Training involves patch extraction and training of the BRAVE-NET architecture. Patch-wise predictions are used during training for performance assessment and for the reconstruction of whole-brain predictions after training. (3a) Whole-brain predictions can directly be evaluated by voxel-wise scores or (3b) optionally further refined in post-processing *via* segment washing. For this step, segment information is additionally utilized. (4) Post-processed predictions can be evaluated by both voxel-wise and segment-wise scores.

Anatomical labeling of arteries depends on *a priori* identification (segmentation) of the arteries and artery segments through the extraction of bifurcation points. Our proposed labeling model not only uses raw image information but also makes use of vessel-specific information extracted from *a priori* segmentation of the vessel tree. In the following, we describe how we prepared our data to have the correct input characteristics. Importantly, all models presented are agnostic to these preparation steps as long as the correct input format is provided. Thus, other data preparation and segmentation procedures are compatible with the presented models. An illustration of our data processing pipeline is shown in Figure 1. Image and vessel-specific information realize our models' input and thus are essential for making predictions (filled black arrows). However, segment information is only required for ground-truth creation (orange arrows) or optionally for post-processing of model predictions (dashed black arrows).

##### Image and vessel-specific information

Time-of-flight magnetic resonance angiography images were utilized in two ways. First, raw scans coming directly from the scanner, i.e., without any common post-processing steps like non-uniformity correction or brain extraction, were gathered. The raw image intensities served as the primary input to our models, termed image input.

Second, vessel centerlines with the corresponding radius at a certain voxel coordinate were extracted, involving two processing steps: (1) we utilized the brain vessel segmentation ground-truth from Hilbert et al. (5), yielding from a semi-automated procedure described in the Data Labeling section of Hilbert et al. (5). This way we aimed to ensure highest quality of input for anatomical labeling; however, we note that this step is directly replaceable by predictions of the BRAVE-NET segmentation model, which would yield comparably accurate segmentations. In particular, cerebral arteries constituting the circle of Willis and the major brain-supplying arteries, such as the internal carotid arteries, the vertebral arteries, and the basilar artery were labeled. (2) Segmentations were thinned into a one voxel thin centerline with the radius values encoded in the voxel value by the implementation of the technique described in detail in Selle et al. (18).

Both image and vessel-specific input values were scaled between 0 and 1, using min–max scaling with training set statistics.

##### Segment information

Additionally, the resulting centerline representation was used to extract segment information. Vessels were divided into segments based on bifurcation points, so that each voxel between two bifurcations gets assigned to the same segment. This step was implemented to facilitate ground-truth labeling as part of the in-house software, described previously in Frey et al. (19). A list of segments with corresponding coordinates yields from the skeletonization algorithm of Selle et al. (18). While it is a necessary step for ground-truth creation, retrieving predictions from the model does not depend on this step, as shown in Figure 1. However, we show a way to utilize segment information *via* an optional post-processing technique—segment washing—, to improve the consistency of voxel-wise predictions of any model. This shows a potential step toward graph-based approaches, where segment information is directly ingrained in the method and is a natural prerequisite.

#### Data labeling

Ground-truth labels of the arterial brain vessel segments were generated semi-manually using a standardized pipeline that has been described previously (19). Briefly, using the aforementioned segmentation, centerline and segment information vessels were labeled using an in-house graphical user interface dedicated to the anatomical labeling of vessels by OUA (5 years of experience in stroke imaging and vessel labeling), EA (5 years of experience in stroke imaging and vessel labeling), or JB (4 years of experience in stroke imaging and vessel labeling). All created ground-truth labels were consecutively cross-checked by another rater. During the labeling process, around 80% of the included patients were found to have missing arterial segments due to pathological variation.

**Figure 2:**
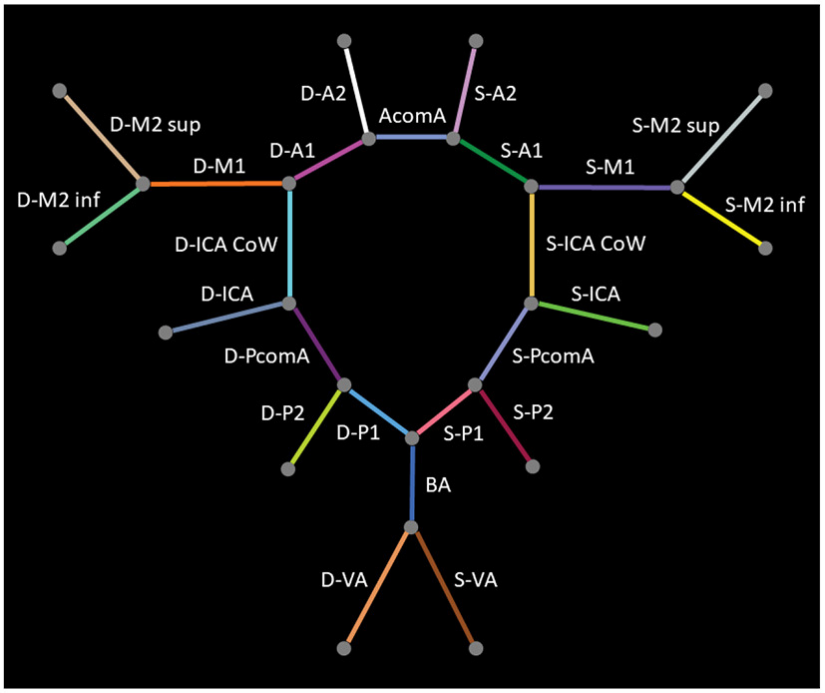
Schematic illustration of intracranial arteries included in our analysis.

In a first step, 24 classes of vessel segments were labeled, with background and non-annotated vessels leading to 26 classes in total: internal carotid artery (ICA), internal carotid artery Circle of Willis segment (ICA CoW), middle cerebral artery first segment (M1), middle cerebral artery second segment superior (M2 sup), middle cerebral artery second segment inferior (M2 inf), anterior cerebral artery first segment (A1), anterior communicating artery (AcomA), anterior cerebral artery second segment (A2), vertebral artery (VA), basilar artery (BA), posterior communicating artery (PcomA), posterior cerebral artery first segment (P1), and posterior cerebral artery second segment (P2). The arteries in the left (Sinister, S-) or right (Dexter, D-) hemisphere are distinguished, except for the unpaired AcomA and BA. Figure 2 provides a schematic illustration of the intracranial arteries of interest for our analysis. We refer to this constellation of segments as detailed in the following.

In a second step, several classes were aggregated into clinically relevant groups resulting in 11 vessel classes in addition to the background non-annotated vessels, hence 13 classes in total: D/S-(ICA, ICA CoW), D/S-(M1, M2 sup, M2 inf), (D-A1, D-A2), (S-A1, S-A2, AcomA), BA, D/S-VA, and D/S-(PcomA, P1, P2). This constellation of segments is termed as aggregated in the following.

#### Cross-validation and patch extraction

We employed a 4-fold cross-validation methodology to ensure the robustness of all models toward different training and evaluation sets. Data were split with random sampling into four distinct training and test sets with no overlapping test patients. The final number of patients was 182 and 60 in each training and test set, respectively; including equal numbers of Pegasus, as well as 1000Plus patients across folds. The random patient selections into folds defined by the framework were saved to ensure all models were trained with the same training sets and evaluated with the same test sets.

Deep learning solutions to voxel-wise classification tasks—especially in the field of medical imaging—are frequently realized in a so-called patch-wise paradigm for two main reasons. Namely, training of DL models for image processing on whole-brain volumes at once demands significant computational resources and requires numerous images. Thanks to the voxel-wise labeled ground-truth, one can reformulate and relax the classification task to an arbitrary neighborhood of any voxel, called an image patch. This way training networks mean less computational burden and as the image of a single patient can yield multiple patches, the number of training samples is increased.

In our case, TOF images and the extracted arterial centerlines with radius values were used as model input. We extracted 3D image and centerline patches together with corresponding ground-truth patches around artery voxels for training. For each arterial segment, we randomly sampled eight voxel coordinates and extracted patches around them in two sizes: 128 × 128 × 64 voxels and 256 × 256 × 128 voxels. The maximal number of available voxels was used in patients where less than eight voxels were labeled as a certain segment. As centerlines were generated from the same TOF image, the resulting image and centerline patches corresponding to a certain voxel are naturally aligned and centered on the same coordinate.

#### Data augmentation

A common practice for increasing model performance and generalization in the field of deep learning is data augmentation by various transformations. This can aid the applicability and generalization of models in cases of yet unseen input variations, hence in the real-world application as well. As an additional experiment, we augmented the training patches by rotations in random axis and random angles in the range from −60° to +60°. The training dataset was inflated three times, i.e., original patches were kept and randomly transformed two times additionally.

### Model architecture

Our proposed architecture is the BRAVE-NET multi-scale segmentation model, developed originally for arterial vessel segmentation (5). BRAVE-NET brings an intuitive extension to the widely used the Unet architecture (20), namely the parallel extraction of low and high-resolution features around a certain voxel. The added context path operates with a larger receptive field down-sampled by half, which allows the identification of the spatial context of a given vessel, lost due to patch-wise training. To feed the standard encoder and context path of the network, patches of two sizes—centered around the same voxel—are required as input.

**Figure 3:**
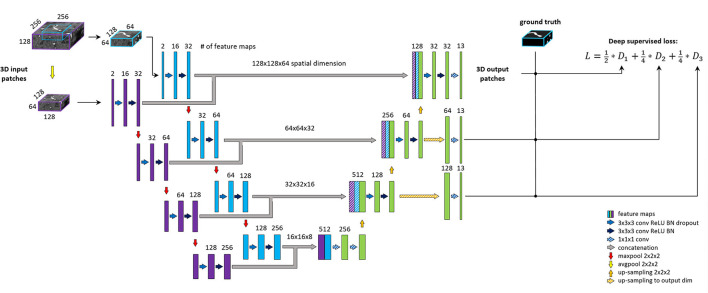
BRAVE-NET architecture. Detailed explanation can be found in Hilbert et al. (5).

Compared to 5, the spatial input dimensions of the network were changed to 128 × 128 × 64 and 256 × 256 × 128 voxels for the original encoder and context path, respectively. Channel dimension—i.e., feature dimension—of the input layers was increased to two so that the network can process image and vessel-specific features together. Due to the increased spatial dimension of patches, we lowered the number of convolutional filters in the first level to 16; consequently, all consecutive levels operate on half the amount of feature maps. For regularization, we found the L2 norm more effective than the dropout, thus removing all dropout layers from the network and applying 1e-3 L2 norm on convolutional weights. The output of the network was also adjusted—including deep supervision outputs—to yield labeling of the 26 or 13 arterial segment labels for the detailed and aggregated vessel constellations, respectively. All other parameters of the BRAVE-NET architecture were kept intact. The architecture is depicted on Figure 3.

### Model training and evaluation

#### Experimental setup and training scheme

Input patches of size (128 × 128 × 64) and (256 × 256 × 128) with image and vessel-specific features with ground-truth label patches of size (128 × 128 × 64) were used to train our models. Models were implemented in the Python programming language using the TensorFlow deep learning framework (21) and trained on a high-performance deep learning workstation using a single NVIDIA Titan RTX GPU. We used the Adam optimizer (22) with an initial learning rate of 1e-3 and categorical cross-entropy loss function. The weight in convolutional layers was initialized using the Glorot uniform initialization scheme (23).

We compared our proposed DL-based labeling network to a classical machine learning baseline, namely a random forest (RF) classifier trained and evaluated on voxels sampled from the same training and test sets of patients across all folds. Training sets for the RF contained an equal amount of voxels per segment label sampled from each patient. We used 50 estimators in the RF model.

Models were trained and evaluated separately for aggregated and detailed vessel constellations, while considering the same patients in the training and test sets of the cross-validation framework.

#### Segment washing

We formulated anatomical labeling of arterial brain vessels as a voxel-wise classification task, where each voxel in a 3D image separately gets assigned to one of the vessel segment labels by the model regardless of the voxel's location. Thus, the output of trained models can naturally contain multiple predicted labels in a single anatomical vessel segment. While this does not impede the visualization of model output, the interpretation might be challenging in case of a high degree of heterogeneity. As Unet-based segmentation models are known to have great local integrity, this phenomenon is mostly expected in areas near bifurcation points, where the network has to assign different labels to voxels with seemingly similar characteristics.

Segment information—i.e., bifurcation information—along the vessel tree, however, can offer a straightforward adjustment of voxel-wise predictions. For this purpose, we developed the following post-processing technique, called segment washing. Segment information was translated into a 3D map of the same dimensions as the TOF-MRA image of the patient. In this map, each voxel between two bifurcation points was assigned the same segment ID. Using this mapping on the predictions, the Softmax scores within a segment were summed up per segment and the label with the highest total Softmax score was assigned to all voxels within the segment. In other words, this procedure washes each segment through with the label of the highest average likelihood and yields homogenous labeling within each segment along the vessel tree.

#### Performance evaluation

To measure class-specific performance of models, we used primarily the F1 score, defined as the harmonic mean of precision and recall, ranging from 0 to 1. Scores were calculated per segment label and per segment group in case of the detailed and aggregated vessel constellation, respectively. To evaluate the overall performance, macro F1 score (mF1) and balanced class accuracy (bAcc) were computed by averaging the class F1 scores and class recalls, respectively, overall vessel segment labels.

Using the above-mentioned metrics, we approached the evaluation of model performance from two angles. First, voxel-wise performance scores were computed from the direct model outputs. This way the exact performance of the proposed DL models is evaluated on the level of voxel classification. However, as longer segments encompass more centerline voxels, voxel-wise performance scores are skewed toward the identification of longer segments. Moreover, to enable a more practical interpretation of the performance, the assessment of anatomical labeling on a segment level can be considered. Hence, second, we included the evaluation of segment-wise classification performance to adhere to better clinical interpretation. Segment-wise scores were calculated from post-processed outputs with segment washing. All voxel-wise predictions in a given segment were pulled together and counted as a single prediction associated with the segment. Thus, these scores are not tampered with by the variability across segment sizes.

Moreover, we evaluate performance per vessel segment labels considered in the detailed and aggregated vessel constellations by class-specific F1 score. Here, we categorize performance as excellent (>0.9), good (>0.75), moderate (>0.6), and poor (<0.6) to allow better overall interpretation.

All evaluation metrics were calculated on whole-brain labels, reconstructed from patch-wise predictions of the models in case of both aggregated and detailed vessel constellations. Background and non-annotated classes were excluded from the calculation of the metrics. We report mean values and standard deviation across test sets defined by the cross-validation framework. To cope with the different output dimensions of the network and the ground-truth dimension, we did not consider the last slice of network output when calculating the metrics.

## Results

In the following experiments, we trained three models in both aggregated and detailed vessel constellations: (1) a random forest classifier (RF), (2) our proposed BRAVE-NET architecture, referred to as proposed, and (3) our proposed BRAVE-NET architecture with additional augmentation of training data, referred to as proposed-augmented. The contribution of segment washing was assessed by applying this step consecutively to the predictions of a certain model; no additional model training was performed.

On a voxel level, most deep learning approaches significantly outperformed the random forest classifier. The proposed BRAVE-NET architecture performed similarly to the standard Unet architecture, but slightly outperformed it with a macro F1 score of 0.89 and a balanced class accuracy score of 0.90 for aggregated and 0.80 and 0.83 for detailed segment classes (RF mF1 of 0.54, 0.34 and bAcc of 0.77 and 0.57, respectively). While comparing various inputs, we can see that the combination of image and vessel-specific information yielded the highest performance. The addition of the segment washing step yielded notable improvement. Augmentation of training data led to a smaller improvement, however, seemed to deliver comparable benefits as segment washing on top of the proposed model with image and vessel inputs. Detailed results are shown in Table 1.

**Table 1:** Voxel-wise test results of the random forest classifier (RF), a Unet, the BRAVE-NET networks, referred to as proposed and BRAVE-NET networks trained with augmented training data, referred to as proposed-augmented.

**Voxel-wise scores**			**Aggregated segments**		**Detailed segments**	
**Model**	**Input**	**Segment washing**	**mF1**	**bAcc**	**mF1**	**bAcc**
RF	Image + vessel	–	0.54	0.77	0.34	0.57
Unet	Image + vessel	–	0.83	0.83	0.71	0.73
Unet	Image + vessel	✓	0.86	0.85	0.75	0.77
Proposed	Image	–	0.50	0.47	0.40	0.40
Proposed	Image	✓	0.63	0.55	0.47	0.45
Proposed	Vessel	–	0.79	0.80	0.68	0.72
Proposed	Vessel	✓	0.84	0.84	0.74	0.76
Proposed	Image + vessel	–	0.84	0.84	0.73	0.76
Proposed	Image + vessel	✓	0.88	0.88	0.78	0.80
Proposed-augmented	Image + vessel	–	0.86	0.87	0.76	0.79
Proposed-augmented	Image + vessel	✓	**0.89**	**0.90**	**0.80**	**0.83**

Results are shown for models trained on aggregated and detailed vessel constellations and subsequent application of segment washing of predictions is indicated. Metrics shown are macro F1 score (mF1) and balanced class accuracy (bAcc). Performance evaluation of the proposed models with segment washing did not involve additional training or retrieval of predictions. Bold values indicate overall best performance with respect to the given metric.

Similarly, on a segment level, the models trained with augmented data yielded higher performance, with a macro F1 score of 0.85 and a balanced accuracy of 0.88 for aggregated, and 0.78 and 0.82 for detailed vessel cases, respectively. Here, all results are reflective of predictions post-processed with segment washing, as this step is naturally required to compute segment-wise scores. The segment-wise performance scores of the models appeared only slightly worse than their voxel-wise scores. Detailed results are shown in Table 2.

**Table 2:** Segment-wise test results of the trained BRAVE-NET networks on aggregated and detailed vessel constellations.

**Segment-wise scores**	**Segment washing**	**Aggregated segments**		**Detailed segments**	
		**mF1**	**bAcc**	**mF1**	**bAcc**
Proposed	✓	0.84	0.86	0.75	0.78
Proposed-augmented	✓	**0.85**	**0.88**	**0.78**	**0.82**

Scores were computed using the same predictions as in case of voxel-wise scores. Bold values indicate overall best performance with respect to the given metric.

**Figure 4:**
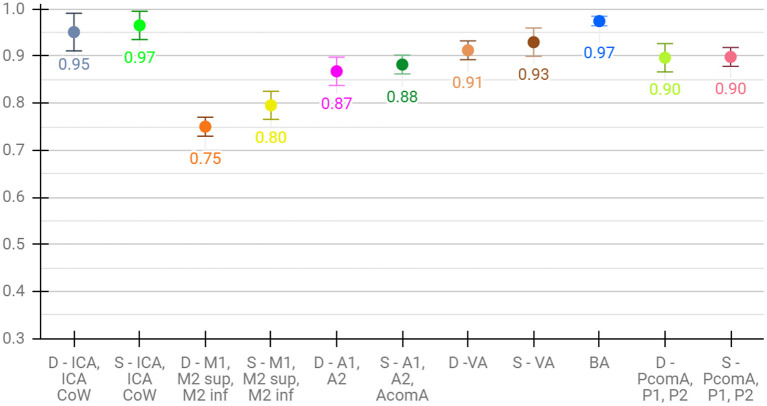
Detailed test results of the proposed-augmented model trained on aggregated vessel constellation with subsequent segment washing, values reflect mean voxel-wise macro F1 scores from cross-validation, error bars correspond to standard deviation across folds.

**Figure 5:**
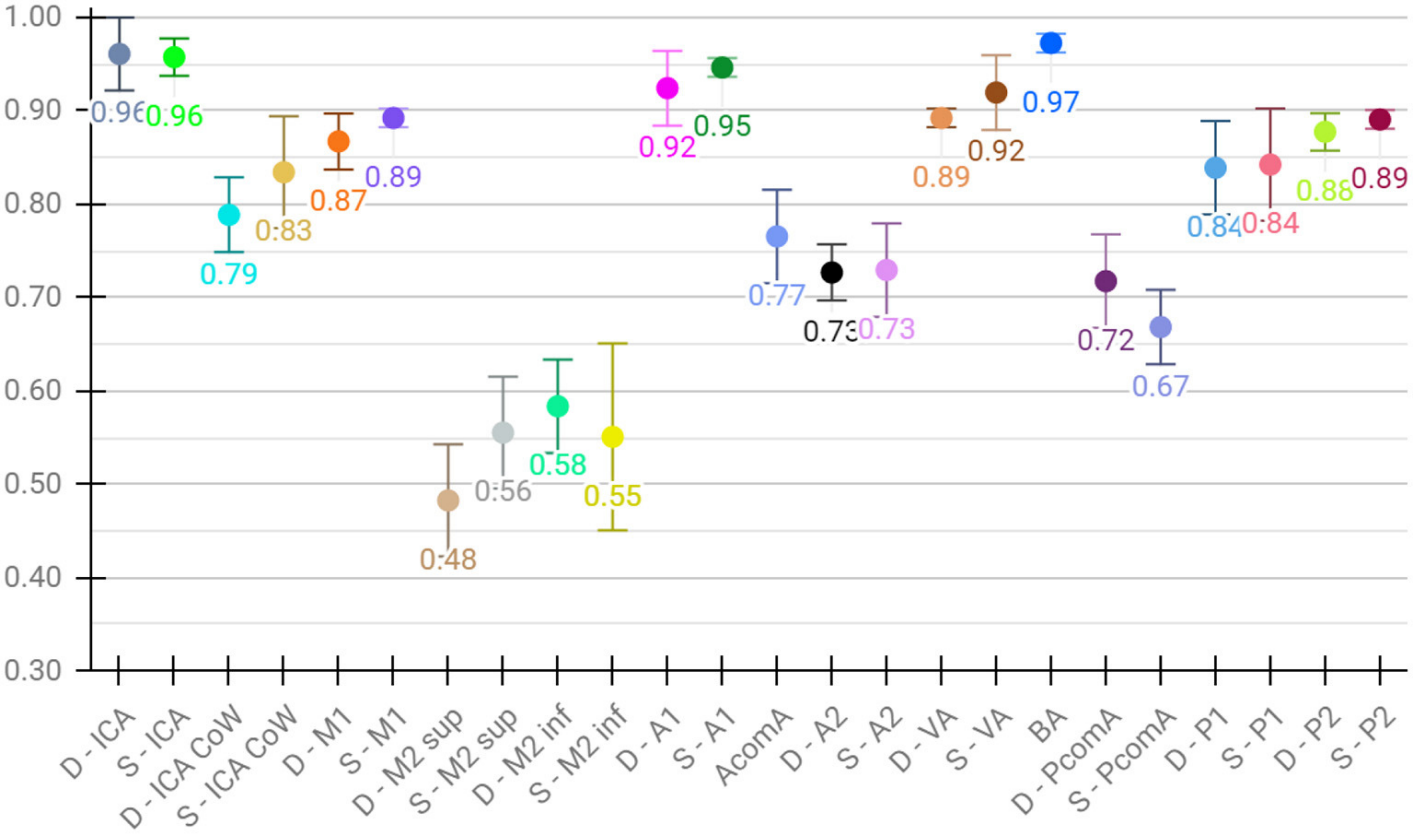
Detailed test results of the proposed augmented model trained on detailed vessel constellation with subsequent segment washing; values reflect mean test voxel-wise macro F1 scores from cross-validation, error bars correspond to standard deviation across folds.

Lastly, for the evaluation of per class performance, we report the mean F1 score across test sets for each vessel segment, both for aggregated (Figure 4) and detailed vessel constellations (Figure 5). Here, results are shown for the best, proposed augmented models with subsequent segment washing on predictions. In case of aggregated segments, seven out of 11 class labels yielded excellently and four good performances, with the group of dexter M1, M2 sup, and M2 inf just meeting the criteria. In case of detailed segments, six out of 24 class labels yielded excellent, 10 good, four moderate, and four poor performances. The four poor labeling results belonged to the left and right M2 sup and M2 inf segments. Moreover, the BRAVE-NET architecture performed similar or better than the standard Unet in all vessel segments and significantly better in the smaller segments; figures are provided in Supplementary material.

**Figure 6:**
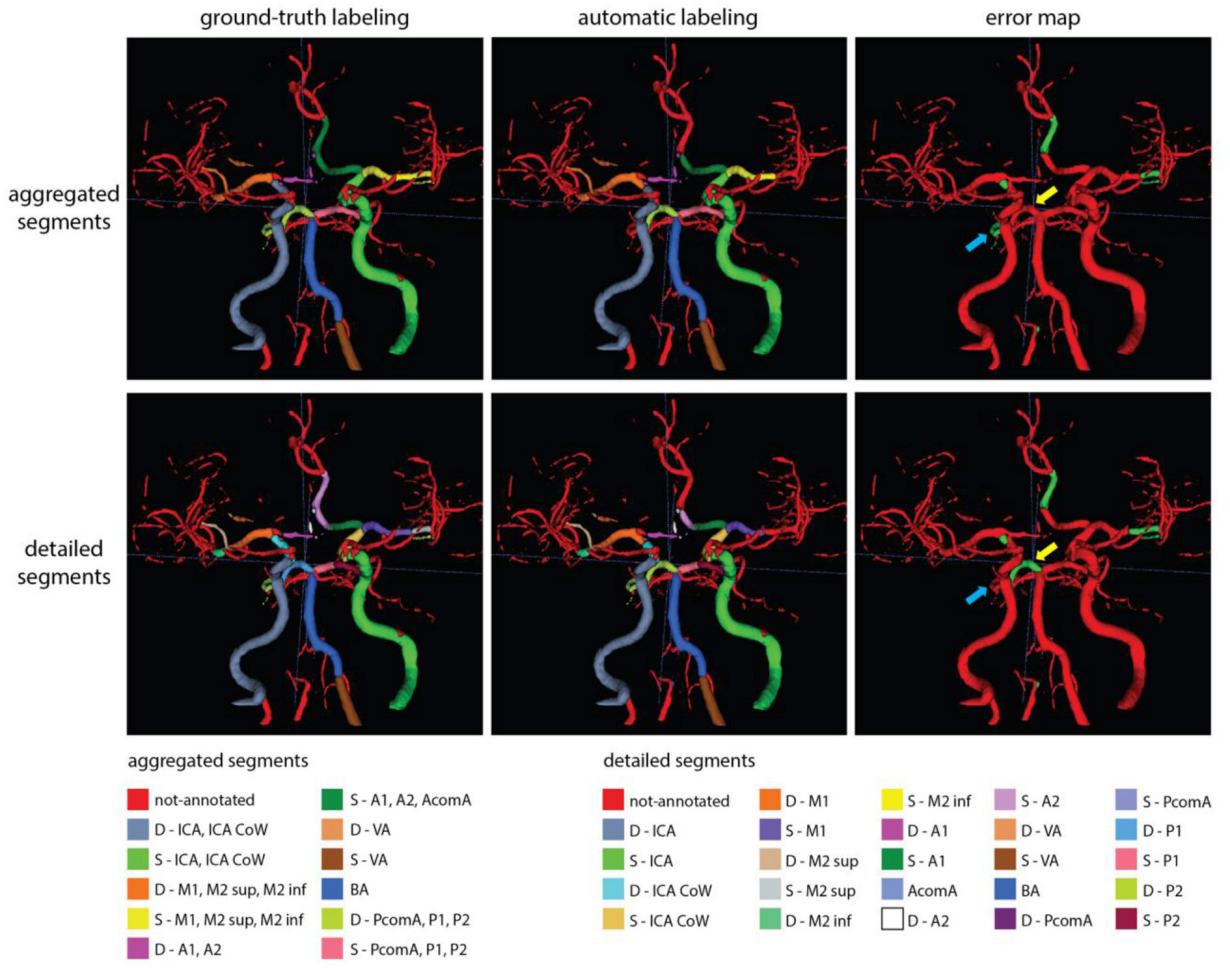
Exemplary test patients with D-P1 misclassified on the detailed segment prediction as D-P2, while correctly classified in the aggregated segment case (yellow arrow). Additionally, while the model trained with detailed segments correctly annotated the D-P2 segment, the model with aggregated segments left it out from the (D-PcomA, P1, P2) group (blue arrow). Model performance in case of this particular patient was 0.89 and 0.80 mF1 for the aggregated and detailed vessel constellations, respectively. Misclassification is shown with green on the error map. Small, red patches intersecting a few segments are slight rendering errors and do not originate from incorrect labeling.

**Figure 7:**
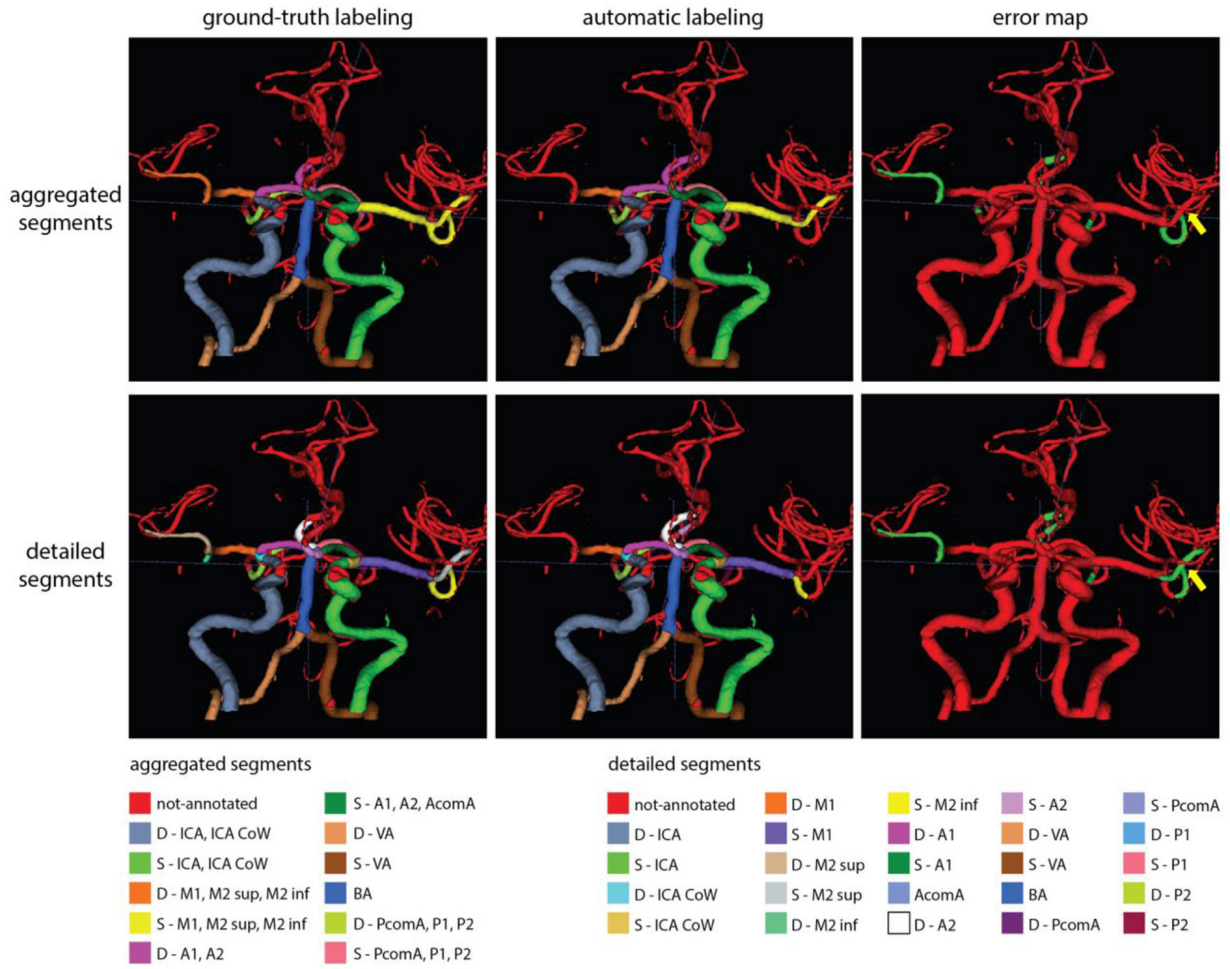
Exemplary test patients with non-annotated S-M2 sup on the detailed segment prediction while correctly classified by the model trained with aggregated segments (yellow arrow). Model performance in case of this particular patient was 0.90 and 0.79 mF1 for the aggregated and detailed vessel constellations, respectively. Misclassification is shown with green on the error map. Small, red patches intersecting a few segments are slight rendering errors and do not originate from incorrect labeling.

Figures 6, 7 show two example test patients with ground-truth and aggregated and detailed segment predictions of our best model with segment washing. Model performance for both aggregated and detailed constellations was comparable to the reported average performance. Error maps show misclassification in green regardless of the true label class. The color of visualized segments corresponds to coloring in Figure 4 for aggregated segments and Figures 2, 5 for detailed segments, respectively.

Automated labeling of a patient's vessel tree took 8 and 10 s with our proposed networks in the aggregated and detailed cases, respectively, using the described GPU system.

## Discussion

We adapted a state-of-the-art, multi-scale deep learning approach for anatomical labeling of intracranial arteries that uses a novel combination of image and vessel-specific information in patients with cerebrovascular disease. We specifically analyzed the performance of networks trained with single inputs and have shown superior performance when combining image and vessel-specific information. Moreover, we gave a comprehensive overview of the necessary processing steps to realize fully automated labeling of intracranial artery segments from raw TOF-MRA. We highlighted which of these steps deem essential for model training and automated labeling and which serves as optional improvement of predictions. Our proposed model achieved high average performance in labeling aggregated, as well as detailed artery segments validated by multiple metrics. The model showed high performance in labeling most arteries of clinical interest in cerebrovascular disease. The presented analysis was conducted in an extensive patient cohort with vessel pathologies which further emphasizes the robustness and applicability of our results to an important clinical need. In our realized pipeline, each necessary processing step can be automated by publicly available sources making our approach highly suited for further clinical testing.

### Comparison to literature

In Table 3, we provide an overview of the necessary pre-processing steps, number of patients in the study, number of arterial segments considered, and best execution times reported by existing literature and compare them to our developed models. Since the anatomical labeling paradigm used in our study and previous works are conceptually different (voxel-wise segment labeling vs. bifurcation classification), we cannot provide a direct comparison of prediction performance.

**Table 3:** Overview of existing methods.

	**Pre-processing**	**No. patients**	**No. segments**	**Execution time**
**Location-based**			
Takemura et al.	re, be, pt	15	12	n.r.
Dunås et al.	re, be	132	14	13 m (CPU)
Shen et al.	re, be, pt	194	9	15.7 s (GPU)
**Graph-based**			
Bilgel et al.	be, tr	30	15	n.r.
Robben et al.	re, tr	50	9	510 s (CPU)
Bogunovic et al.	re, be	50	9	n.r.
Chen et al.	re, be, nm, tr	729	22	0.1 s (GPU)
**Other**			
Zhang et al.	nm, mm	109	9	n.r.
**Proposed**			
Proposed detailed	–	242	24	10 s (GPU)
Proposed detailed + segment washing	be	242	24	10 s (GPU)
Proposed aggregated	–	242	11	8 s (GPU)
Proposed aggregated + segment washing	be	242	11	8 s (GPU)

Methods are compared in terms of required pre-processing steps from the raw image to produce the proposed anatomical labeling (pre-processing), total number of patients included in the study (number of patients), number of arterial segments considered and labeled by the model (number of segments), and best execution time reported by authors (execution time). Due to being a prerequisite of each method, vessel segmentation and centerline extraction are omitted from the list of required pre-processing steps. re, registration; be, bifurcation extraction; pt, additional reference point tracking (Takemura et al.—tracking of center of CoW, Shen et al.—key point tracking); tr, spatial transformation (Robben et al.—scale-space transformation, Chen et al., Zhu et al., and Bilgel et al.—isotropic reslicing); nm, intensity normalization; mm, mesh modeling; nr, not reported.

First, vessel segmentation and centerline extraction are an essential prerequisite of all but one work (8), where only vessel segmentation was employed. These steps are rather straightforward, well-researched, and automated solutions exist, hence we exclude them from our comparison.

While location-based methods rely by nature on co-registration to the template atlas, only (9) succeeded to circumvent this step from the graph-based group. Similarly, bifurcation extraction is a natural prerequisite for graph-based methods and seemed to be an essential element for all but one reviewed work (12). Both of these steps help a great deal in localization and precise distinction of arterial segments, however, rely heavily on image quality, might involve exhaustive computations, and can be adversely affected by pathologies or unusual anatomy—e.g., missing arterial segments. Furthermore, four out of the five reviewed, graph-based works required some sort of spatial transformation of the images prior to labeling. Robben et al. (12) employed scale-space transformation to 4D to model vessel radius variation whereas Bilgel et al. (9), Chen et al. (11), and Zhu et al. (13) applied resampling of the images to isotropic voxel sizes. While scale-space representation can enhance modeling capacity, isotropic resampling can introduce noise or severely reduce resolution (depending on down/up-sampling) prohibiting good performance in case of highly distinct voxel sizes. Lastly, the method of Zhang et al. (24) depended on mesh modeling and standardization of the 3D mesh due to prerequisites of the applied MeshCNN network. In contrast, the herein presented DL network clearly relies on the least amount of pre-processing. Thanks to the translational equivariance property of convolutional layers there is no need for co-registration, identification, and learning of local context is realized through the context path of the BRAVE-NET architecture, while full 3D convolutions enable learning of anisotropic voxel distances by nature. Through voxel-wise classification, we alleviated the need for bifurcation extraction and showed the flexibility of our proposed pipeline in incorporating such information through segment washing for a slight performance increase. While the post-processed predictions yielded superior performance, the difference compared to the proposed model trained with augmented data remained small. Moreover, predictions as well as bifurcation information about a certain segment are independent of the preceding and following segments. This means, neither our deep learning approach nor the segment washing post-processing technique can be flawed by missing segments.

Second, the number of patients employed in the reviewed studies varied. There were five works—beyond ours—that utilized more than 100 patients. To show clinical applicability, methods need to provide thorough evaluation including a great variety and number of patients. Our work comes second in this aspect, employing two pathology-rich datasets of cerebrovascular patients. However, important to mention that even though our reported evaluation was conducted on whole image volumes, our networks were trained patch-wise, enabling them to see an increased number of local variations of arteries (roughly 30.000 and 16.000 training patches excluding augmentation in case of detailed and aggregated segments, respectively).

Third, we observed a similar variation in terms of the considered arterial segments for labeling. Most works—except (11, 13)—restricted their models to a significantly smaller portion of intracranial segments, which clearly limits clinical applicability. Besides being able to process and label most segments, our model can naturally adapt to any arbitrary scenario where aggregated groups of arterial segments are of clinical interest, without the need of redefining computational graphs or prior knowledge bases.

Fourth, we included execution times in our comparison, owing to the prompt time criteria of most clinical settings. We found two among the reviewed works (7, 11) that reported similar computation times to our method, both corresponding to predictions in a GPU system. Due to the widespread availability of GPU technology we do not consider this a higher requirement than a modern CPU system. We deem near—or lower than—15-second computational times per whole volume labeling adequate for real-time clinical usage; thus, we position our method together with (7, 11) on top of the list from this aspect. We would like to note, however, that the reviewed and present study did not use the same computational resources. This means differences to a certain degree are natural and data are mainly provided for a more complete overview of the reviewed works.

In summary, due to outstanding advantages in regard to necessary pre-processing, sufficient training and evaluation cohort to enable good generalization and ability to learn pathologies, great flexibility in adapting to various clinical definitions of arteries of interest, and fast prediction times, we claim our solution superior in the applicability for clinical setting compared to other methods in the literature.

### Anatomical labeling in other vascular structures

Since deep learning, i.e., data-driven solutions depend primarily on the structure of data they were designed for, methodologies can apply across different medical specialties. Beyond intracranial arteries, there has been methodologically relevant research in anatomical labeling of the vasculature of other organs (25–28). Recently, Yang et al. proposed an encouraging and promising solution to anatomical labeling of coronary arteries (29). Motivated by the limitations of purely graph-based methods, the authors proposed a hybrid framework combining a graph convolutional network with a condition extractor mechanism that extracts 3D spatial image features along the branches by a 3D convolutional neural network and long short-term memory model. Their sophisticated method demonstrates another way of extending graph-based methods by the image processing capability of DL. Even though intracranial arteries tend to have a bigger variety of tortuous vascular structures than cardiac arteries, we note the potential applicability of such hybrid methods in cerebrovascular labeling.

Our proposed post-processing step, segment washing points toward such a hybrid direction. Taking it further, a hierarchical refinement as a second step after aggregated prediction or a combined hierarchical loss function could help to advance performance in more distal segments. As shown in our results, our detailed vessel model suffered from limited performance on the smallest, more tortuous branches, namely the M2 sup and inf segments. Figure 7 shows a perfect example of potential improvement, where the M2 sup segment was correctly captured by our model trained with aggregated segments but was misclassified as non-annotated by the model trained on detailed segments. Information exchange between the two models about the M2 sup segment belonging to the MCA branch could have prevented the detailed model from not labeling the given segment.

### Clinical aspect

In the clinical setting, arterial brain vessels are routinely imaged and there is a major clinical need to translate the information contained within these images into utilizable biomarkers. This quantifiable information can potentially be applied for the prediction of disease, disease progression, and response to treatment (3). For example, in chronic cerebrovascular disease, this information could inform monitoring of the disease and the prediction of cerebrovascular events. In acute stroke, existing and potentially missing vessels could be flagged. In addition, these tools could also facilitate the treatment of acute stroke in settings, where specialized neurologists and radiologists are not available. Here, our model which does not require post-processing and could be applied on the scanner console is a promising proof of concept for the future development of arterial biomarkers. The segmented and anatomically labeled vasculature can be measured and the values can be translated into scores. Or, the information can be utilized directly for data-driven predictive modeling.

Within this context, a major strength of our study is the utilization of imaging from patients with vessel pathologies owing to cerebrovascular disease. In contrast to healthy patients, cerebrovascular disease leads to vascular changes, such as caliber changes, steno-occlusions, and missing segments. These changes add to the already considerable heterogeneity of the vasculature under healthy conditions. Here, our results are highly promising as we achieved an overall high performance in this patient cohort. However, in some areas like the M2 segment, we found considerably lower performance in contrast to other segments. We hypothesize that this is related to the variations in the localization of the MCA rather than specific MCA variants. MCA variants like fenestrations and duplications are relatively rare (30). However, the MCA is the phylogenetically youngest major brain artery system and it evolved together with the growing frontal, parietal and temporal lobes (31). This allows the MCA to have a high degree of localization freedom in contrast to the more constrained anatomical localization of the phylogenetically older ACA and PCA. Given the clinical importance of the MCA vessels, e.g., for stroke, the performance in the MCA regions needs to be improved. If our hypothesis holds true, this issue can be mitigated by training future models with even larger amounts of patients capturing more localization variations of the MCA. Also here it will be important to include a sufficient number of patients with pathologies of the vasculature, as well as natural variations.

### Limitations

Our study has recognized limitations. First, the generalization of DL methods highly depends on the data source. While the datasets in our study exhibited a large variety of cerebrovascular disease, we recommend specifically including datasets with other pathologies, other MR scanner- and imaging sequence types and other imaging modalities, such as CT, in future works. Second, due to high resource demand, no systematic visual assessment of our results was conducted. We used a 4-fold cross-validation framework to ensure the robustness of our results, which—combined with our extensive dataset—creates an extremely time-consuming environment for visual review. Extensive quantitative evaluation provides a concise overview of our model's strengths and weaknesses; however, understanding model behavior in more detail by visual assessment cannot be ruled out. Third, we have seen limited performance in the smallest artery segments of our detailed vessel models, namely in M2 sup and M2 inf segments. Affirmatively, previous works have reported the processing of narrow and tortuous brain vessels by DL methods challenging (5, 15). We believe that this challenge could be mitigated by the previously noted extensions toward exploiting hierarchies between aggregated groups and detailed segments. Another limitation in this context is that we developed our labeling framework in this first iteration for the most common anatomical variant, where the M1 bifurcates into M2 superior and M2 inferior segments. This accounts for around 80% of patients. It is likely that the performance for the M2 segments will increase when trifurcations and the presence of many small vessels will also be accounted for.

## Conclusion

In conclusion, we could demonstrate that deep learning can be utilized with high accuracy for anatomical labeling of intracranial arteries using TOF-MRA images. Our results suggest that the extensive information on image intensities can be efficiently and beneficially exploited. Due to minimal pre-processing, simple usability, and fast predictions, our method has excellent potential with regard to applicability in the clinical setting compared to current approaches shown in the literature. Furthermore, we have shown that the employed state-of-the-art segmentation architecture further excels in the field of anatomical labeling. Owing to the dependence on anatomical labeling and vessel segmentation, this suggests high promises of interconnected implementations for an even faster and more efficient pipeline. We believe our work provides a crucial step toward realizing the promises and impact of AI in the quantification of cerebrovascular information and thus contributes to a solution for an important clinical need.

## Data availability statement

The datasets presented in this article are not readily available because of ethical and privacy restrictions. Requests to access the datasets should be directed to the corresponding author/s.

## Ethics statement

The studies involving human participants were reviewed and approved by Charité Universitätsmedizin Berlin. The patients/participants provided their written informed consent to participate in this study.

## Author contributions

Conceptualization: AH, JR, VM, DF, and ML. Data curation: EA, OA, JB, VM, AK, IG, JF, and JS. Methodology: AH, JR, and ML. Model development, and validation, and writing—original draft: AH, JR, VM, and DF. Writing—review and editing: All authors. All authors contributed to the article and approved the submitted version.

## Funding

This work has received funding from the German Federal Ministry of Education and Research through a GO-Bio grant for the research group PREDICTioN2020 (lead: DF, No. 031B0154), and funding from the European Commission *via* the Horizon 2020 program for PRECISE4Q (No. 777107, lead: DF).

## Conflict of interest

Author AH reported receiving personal fees from ai4medicine outside the submitted work. Author VM reported receiving personal fees from ai4medicine outside the submitted work. Author DF reported receiving grants from the European Commission and the German Federal Ministry of Education and Research, reported receiving personal fees from and holding an equity interest in ai4medicine outside the submitted work. Author JF has received consulting and advisory board fees from BioClinica, Cerevast, Artemida, Brainomix, Biogen, BMS, EISAI, and Guerbet. There is no connection, commercial exploitation, transfer, or association between the projects of ai4medicine and the results presented in this work. The remaining authors declare that the research was conducted in the absence of any commercial or financial relationships that could be construed as a potential conflict of interest.

## Publisher's note

All claims expressed in this article are solely those of the authors and do not necessarily represent those of their affiliated organizations, or those of the publisher, the editors and the reviewers. Any product that may be evaluated in this article, or claim that may be made by its manufacturer, is not guaranteed or endorsed by the publisher.
