# Supplementary material for: Anatomical labeling of intracranial arteries with deep learning in patients with cerebrovascular disease
Source: Front Neurol. 2022 Oct 17;13:1000914. doi: 10.3389/fneur.2022.1000914 (PMC9634733; doi:10.3389/fneur.2022.1000914)
Supplement: Supplementary file 1 [file Data_Sheet_1.docx]

# Appendix: Model card

## Model Details

Developed by the Charite Lab for AI in Medicine (CLAIM) research group at Charite University Hospital, Berlin, person to contact: Adam Hilbert (adam.hilbert@charite.de)

Version: v1.0, 2021

A previously published BRAVE-NET - multiscale 3D Unet architecture with additional encoding path to capture contextual information - has been adapted for anatomical labeling of 24 classes of intracranial artery segments. The spatial input dimensions of the network were adjusted to 128x128x64 voxels and 256x256x128 for the original encoder and context-path respectively. Input layer depth was increased to 2 to accommodate for image and vessel specific input. Convolutional filters in the first level were lowered to 16, consequently, all consecutive levels operate on half the amount of feature maps compared to the original BRAVE-NET model. All dropout layers were removed, 1e-3 L2 norm was applied on convolutional weights. Categorical cross-entropy loss was used to train the model. Deep supervision of decoder layers was applied by upsampling to output dimensions and generating output prediction to be added to the loss.

Hilbert, Rieger et.al. 2021 “*Anatomical Labeling of Intracranial Arteries with Deep Learning in Patients with Cerebrovascular Disease*”


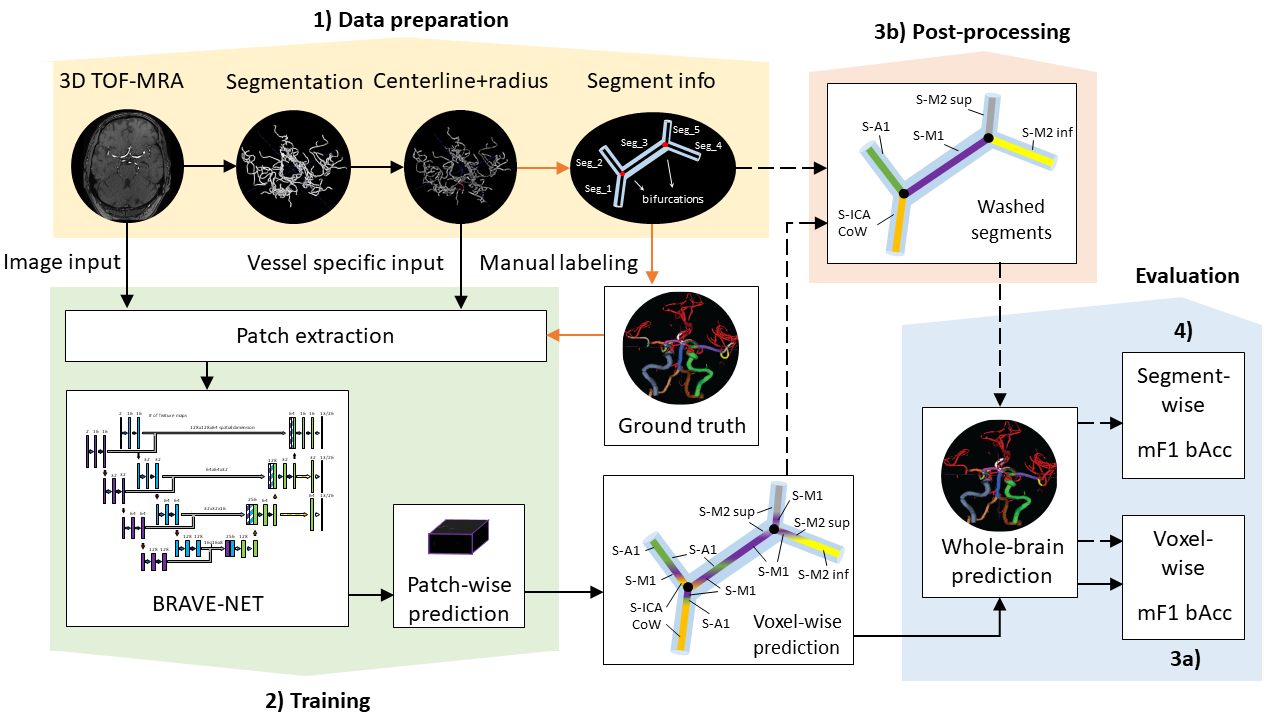


## Intended Use

Automatic anatomical labeling of intracranial arteries in TOF-MRA images.

Accelerating inspection of intracranial arteries, identification of anatomical segments to facilitate diagnosis, monitoring of cerebrovascular disease and to assist intervention. Automated labeling can provide input for other quantitative models to yield potential biomarkers of cerebrovascular status for diagnostic stratification.

The processing pipeline can be applied (model can be re-trained/fine-tuned and evaluated) to the vasculature of other organs as well as to other imaging modalities (e.g. CTA).

## Factors

Relevant factors that might affect performance include groups of patients with different cerebrovascular disease and vessel abnormalities as well as artefacts, noise in imaging and different configurations of scanners. Furthermore, model convergence and performance highly depends on the quality of the presented ground-truth labels, the representation of the included segment classes in training data as well as segmentation and centerline extraction pre-processing steps.

Due to limitation of data, influence of patient groups, pathologies and imaging factors was not evaluated.

## Metrics

Class specific model performance was evaluated primarily by the F1 score. To evaluate overall performance, macro F1 score and balanced class accuracy was computed by averaging the class F1 scores and class recalls respectively over all vessel segment labels. The evaluation metrics were calculated on whole brain predictions (reconstructed from patch-wise predictions). Using these metrics, 1) voxel-wise and 2) segment-wise performance scores were computed. The later requires application of segment-washing post-processing.

Evaluation was done in a 4-fold cross-validation framework. 75% of all data was used for training and 25% for testing in each fold. Average performance and standard deviation across folds are reported.

## Training Data

Retrospective data of 242 patients with cerebrovascular disease from PEGASUS (Martin et al., 2015) and 1000Plus (Hotter et al., 2009) studies was included in total.

Pre-processing included segmentation of vessels and extraction of vessel centerlines with corresponding radius at a certain voxel coordinate to prepare the necessary vessel specific model input. There was no pre-processing applied for raw image input.

Ground truth labeling was done using a standardized pipeline and inhouse software previously described in (Frey et al., 2021), involving the following steps 1) vessels were divided into segments based on bifurcation points, 2) 24 classes of segments were labeled, with background and non-annotated vessels leading to 26 classes in total 3) several classes were aggregated into clinically relevant groups resulting in 11 vessel classes in addition to the background non-annotated vessels, hence 13 classes in total.

Manual labeling was done by OUA (4 years experience in stroke imaging and vessel labeling), EA (4 years experience in stroke imaging and vessel labeling) or JB (3 years experience in stroke imaging and vessel labeling). All created ground-truth labels were consecutively cross-checked by another rater.

Model input comprised of 1) raw image intensities from the TOF-MRA image and 2) centerlines with radius values as vessel specific input. For each segment class 8 voxel coordinates per patient were randomly sampled and patches were extracted around them. In patients, where less than 8 voxels were labeled as a certain segment the maximal number of available voxels were taken.

Training patches were augmented by rotations two times - while keeping the original patch as well - in random axis and random angles in the range from -60° to +60°.

## Evaluation Data

Evaluation was done on whole brain predictions using F1 score and balanced class accuracy metrics. As post-processing, segment washing was done on predictions to achieve best performance.

## Quantitative Analyses

Best results are shown for voxel-wise and segment-wise scores.

|  | Aggregated segments | | Detailed segments | |
| --- | --- | --- | --- | --- |
|  | mF1 | bAcc | mF1 | bAcc |
| DL-augmented (voxel-wise) | 0.89 | 0.90 | 0.80 | 0.83 |
| DL-augmented (segment-wise) | 0.85 | 0.88 | 0.78 | 0.82 |

## Caveats and Recommendations

Regarding the BRAVE-NET architecture, recommendations in the originally published Model card in (Hilbert et al., 2020) should be taken into account.

We note certain caveats in case of clinical application originating from the specific population of patients and pathologies present in the dataset. Our models were trained on TOF-MRA images of patients with chronic steno-occlusive disease (N=72) and acute ischemic stroke (N=170), all registered in Germany. Model performance might differ in case of:

- Patients with other cerebrovascular disease
- Patients of different ethnics
- Different vascular imaging modality (e.g. Computed Tomography Angiography or Digital Subtraction Angiography)
- In presence of further pathologies or neurological disease affecting cerebral vasculature (e.g. aneurysm, Alzheimer disease, multiple sclerosis)
- Different anatomical variants of certain arteries (e.g. M1 trifurcation)

# Appendix: Results


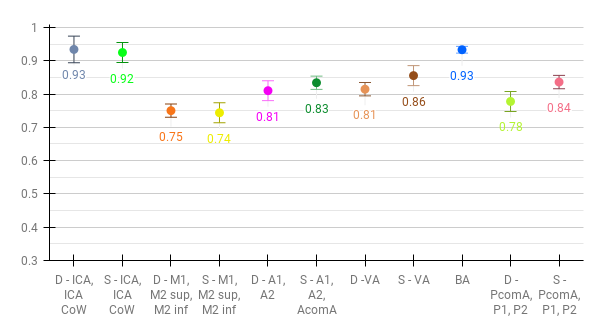


Figure S1. Detailed test results of the Unet model trained on aggregated vessel constellation; values reflect mean voxel-wise macro F1 scores from cross-validation, error bars correspond to standard deviation across folds


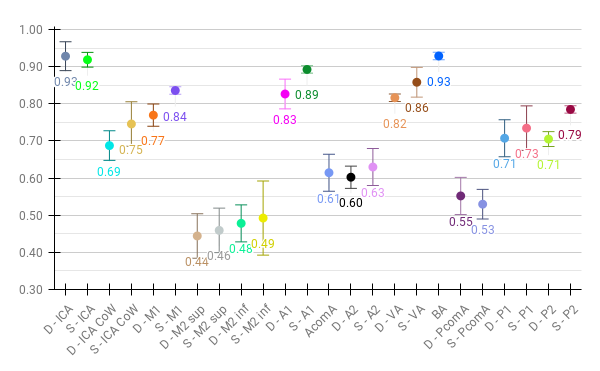


Figure S2. Detailed test results of the Unet model trained on detailed vessel constellation, values reflect mean voxel-wise macro F1 scores from cross-validation, error bars correspond to standard deviation across folds


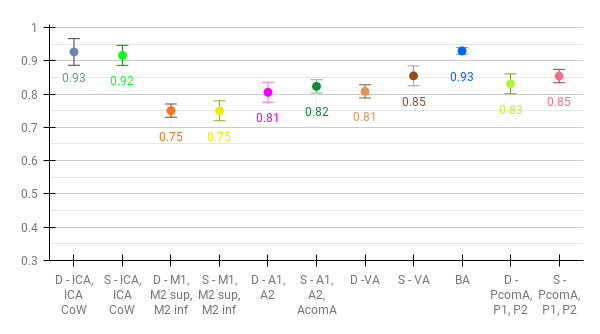


Figure S3. Detailed test results of the Proposed model trained on aggregated vessel constellation; values reflect mean voxel-wise macro F1 scores from cross-validation, error bars correspond to standard deviation across folds


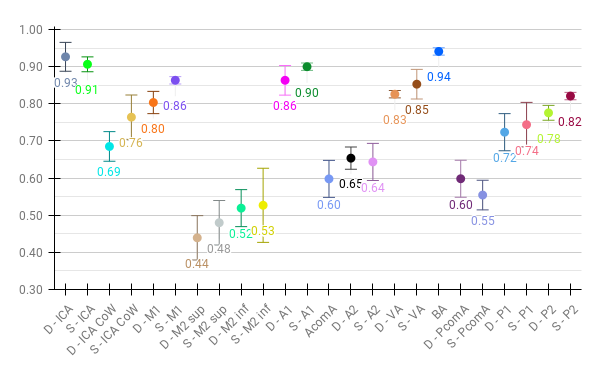


Figure S4. Detailed test results of the Proposed model trained on detailed vessel constellation, values reflect mean voxel-wise macro F1 scores from cross-validation, error bars correspond to standard deviation across folds
